# Mission M-Possible: Mitral Transcatheter Edge-to-Edge Repair in Mitral Annular Calcification Using the Crab-Walk Technique

**DOI:** 10.1016/j.shj.2025.100736

**Published:** 2025-10-29

**Authors:** John Abdelmalek, Muhammad Qudrat-Ullah, Ahmed Souka, Sibi Thomas, Ralph Matar, Lee Hafen, Karim Al-Azizi

**Affiliations:** a Department of Cardiology, Texas Tech University Health Sciences Center, Lubbock, Texas, USA; b Department of Cardiology, University of Kansas Health System St. Francis Campus, Topeka, Kansas, USA; c Department of Cardiology, Baylor Scott and White, The Heart Hospital Plano, Plano, Texas, USA; d Department of Cardiovascular Surgery, Baylor Scott and White, The Heart Hospital Plano, Plano, Texas, USA

**Keywords:** Degenerative mitral regurgitation, Mitral annular calcification, Mitral regurgitation, Mitral transcatheter edge-to-edge repair

## Abstract

**Background:**

Mitral annular calcification (MAC) complicates transcatheter interventions for degenerative mitral regurgitation (DMR), particularly with small mitral valve orifice areas (MVOAs).

**Case Summary:**

An 83-year-old woman with severe DMR, severe MAC, and MVOA of 3.8 cm^2^ presented post-heart failure hospitalization with declining function. High surgical risk and unfavorable transcatheter mitral valve replacement anatomy (neo-LVOT 0.30 cm^2^) limited options to transcatheter edge-to-edge repair (TEER). Using the Edwards PASCAL precision system and the “Crab-Walk” technique (independent leaflet clasp manipulation for MAC-related challenges), TEER reduced mitral regurgitation to mild (1+), with a 2 mmHg gradient at 3 months. The patient was weaned off oxygen and remains asymptomatic at 1 year.

**Discussion:**

This first reported mitral TEER case in DMR with significant MAC and small MVOA highlights the “Crab-Walk” technique’s innovation.

**Take-Home Messages:**

TEER with novel techniques is viable for DMR with MAC when transcatheter mitral valve replacement is prohibitive.

## Highlights

• Mitral annular calcification (MAC) poses significant clinical and technical challenges for heart teams, with surgical interventions carrying high risks and transcatheter mitral valve replacement often contraindicated due to small neo-left ventricular outflow tract areas.
• Mitral transcatheter edge-to-edge repair can be performed in carefully selected patients with degenerative mitral regurgitation and MAC, supported by emerging evidence.
• This case highlights the successful use of the PASCAL precision system with a novel technique, Crab-Walk, to treat severe degenerative mitral regurgitation in a patient with significant MAC and a small mitral valve area (3.8 cm^2^), demonstrating a novel approach to overcome challenging anatomy in MAC-related mitral regurgitation.

## Introduction

An 83-year-old woman presented to our valve clinic for the evaluation of severe symptomatic degenerative mitral regurgitation (MR). She had failed maximally tolerated medical therapy, including diuretics, for 9 months and had become oxygen dependent following a recent hospitalization for heart failure exacerbation.

### Past Medical History

Her medical history included hypertension, hypothyroidism, chronic anemia, chronic kidney disease, chronic thrombocytopenia, biliary cirrhosis (diagnosed in 1980), and cholecystectomy (1981).

### Investigations

**Figure 1:**
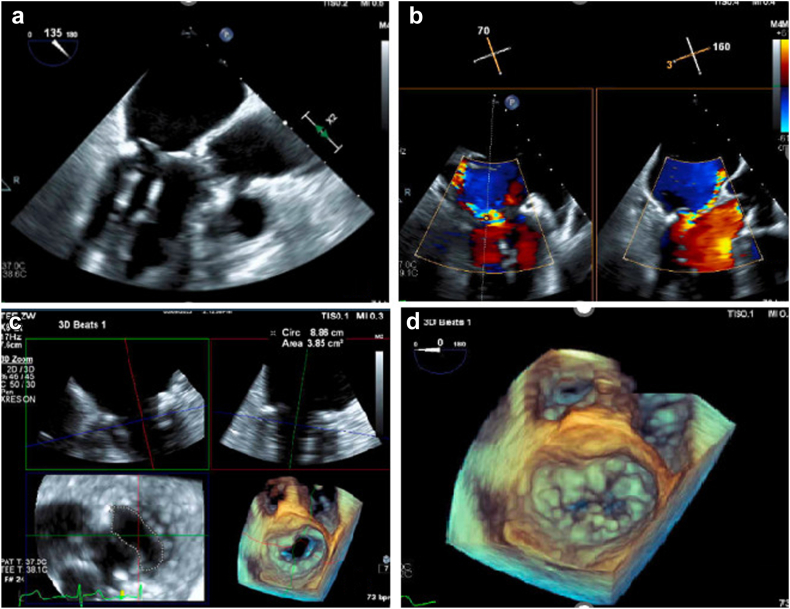
(a) TEE LAX view showing flail P2 segment with torn chordae and MAC extending to leaflets and subvalvular apparatus. (b) Color Doppler in LAX and commissural views showing severe eccentric MR. (c) 3D mitral valve reconstruction showing concentric MAC and valve area of 3.8 cm^2^. (d) P2 prolapse on 3D reconstruction (surgeon's view). Abbreviations: MAC, mitral annular calcification; MR, mitral regurgitation; TEE, transesophageal echocardiography.

Echocardiography revealed calcified degenerative mitral valve disease with significant mitral annular calcification (MAC), mostly posterior, severe eccentric MR with anteriorly directed jet, and a flail P2 segment of the posterior mitral valve leaflet. The mitral valve orifice area (MVOA) was 3.8 cm^2^, and the ejection fraction was 60%–64%, with mild regurgitation in the other valves (Figure 1; Videos 1-4). The patient had persistent New York Heart Association class III symptoms.

### Management

**Figure 2:**
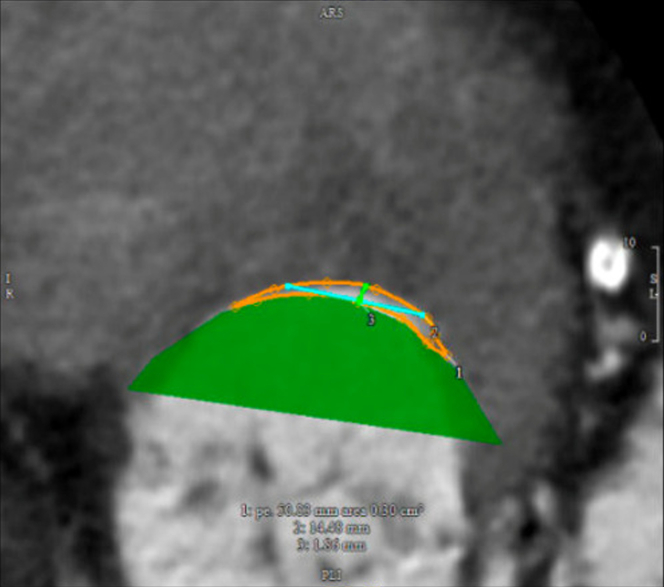
Cardiac CT showing prohibitive predicted neo-LVOT area of 0.30 cm^2^ for TMVR with ViMAC. Abbreviations: CT, computed tomography; LVOT, left ventricular outflow tract; TMVR, transcatheter mitral valve replacement; ViMAC, valve-in-mitral annular calcification.

**Figure 3:**
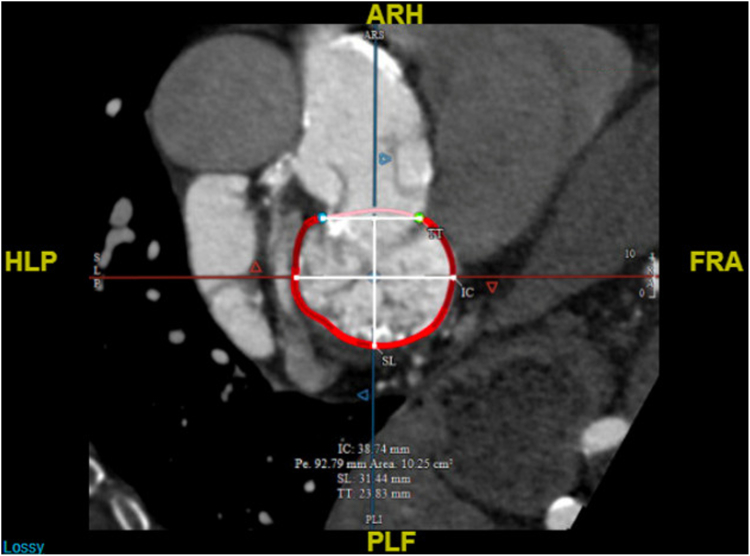
Cardiac CT showing large intercommissural diameter of 38.74 mm for TMVR with ViMAC. Abbreviations: CT, computed tomography; TMVR, transcatheter mitral valve replacement; ViMAC, valve-in-mitral annular calcification.

Given her advanced age, comorbidities, clinical frailty scale of 2/4, Model for End-stage Liver Disease score of 11, and high Society of Thoracic Surgeons scores for mitral valve repair (11.82%) and replacement (13.21%), she was deemed a high surgical risk. She also had an unaccounted-for technical risk which is the severe MAC, putting her at risk for atrioventricular groove disruption, a fatal surgical complication. Evaluation for a percutaneous transcatheter mitral valve replacement (TMVR) trial with valve-in-MAC was unfavorable due to a prohibitive predicted neo-left ventricular outflow tract area of 0.30 cm^2^ and a large intercommissural diameter of 38.74 mm for valve-in-MAC (Figures 2 and 3).

After extensive heart team discussion, transcatheter edge-to-edge repair (TEER) was planned, with the option to abort if severe mitral stenosis developed after device placement but before release. Procedural challenges included significant MAC at the leaflet bases, calcification of the subvalvular apparatus, a small mitral valve area (<4.0 cm^2^), and A2-P2 pathology, increasing risks of high postprocedural gradients, leaflet grasping difficulties, and potential implant migration or dislodgment.

Decision was made to choose the PASCAL Ace implant. Central spacer feature resulting in a larger residual MVOA after TEER was critical to prevent iatrogenic mitral stenosis. Narrower Ace implant with a smaller central spacer as compared with the PASCAL P10 implant was deemed necessary to navigate patient’s small mitral valve area and short leaflet grasping lengths with her complex MAC, although precise and anatomical grasping was the key to prevent leaflet tension and resulting distortion in anatomy and mitral stenosis.

The patient was prepared in the standard fashion. Ultrasound-guided access was obtained in the right common femoral vein. Heparin was administered to achieve an activated clotting time greater than 250 seconds. An 8.5F TorFlex Transseptal Guiding Sheath (Boston Scientific, Marlborough, MA) and a BRK Transseptal Needle (Abbott, Abbott Park, IL) were used to perform the transseptal puncture. Transesophageal echocardiography confirmed adequate height and posterior positioning to align with the medial commissure before crossing the septum. An Amplatz Super Stiff Guidewire (Boston Scientific, Marlborough, MA) was advanced to exchange the transseptal sheath for the 22F steerable guide sheath of the PASCAL Precision Transcatheter Valve Repair System (Edwards Lifesciences, Irvine, CA).

The PASCAL Ace implant, featuring 6 mm nitinol paddles and a 2 mm central spacer, was then delivered via the PASCAL precision system’s steerable catheter.

Initial attempts to position the implant over the flail segment were hindered by significant posterior MAC indentation, causing the device to rotate and engage in “side-bites”. After two unsuccessful attempts, a traditional approach was deemed infeasible, as the patient’s small mitral valve area precluded a second implant due to the high likelihood of inducing mitral stenosis. The goal was precise deployment with a single implant to treat the lesion and reduce MR.

**Figure 4:**
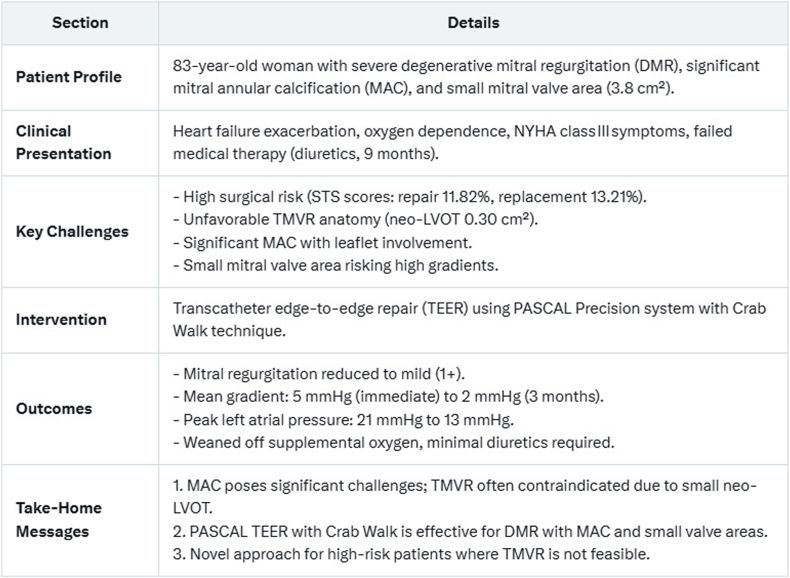
Additional illustrative case figure. Abbreviations: neo-LVOT, neo-left ventricular outflow tract; NYHA, New York Heart Association; STS, Society of Thoracic Surgeons; TMVR, transcatheter mitral valve replacement.

The leaflet capture strategy was modified, and a novel “Crab Walk” technique was implemented as follows “Figure 4”. Initially, the implant’s rotation positioned the anterior clasp laterally on the anterior leaflet and medially on the posterior leaflet in an 11 to 5 configuration within the A2-P2 segment. So, leveraging the independent grasping capability, the clasps were placed in a capture-ready configuration, and the anterior leaflet was released. Using a “towel-twist” maneuver—clockwise rotation of the implant and counterclockwise rotation of the delivery system—the anterior clasp was repositioned medially toward the valve center, achieving a 1 to 7 configuration. The posterior clasp, now the most lateral component, was lifted to free the posterior leaflet. Careful counterclockwise rotation of the implant, paired with clockwise rotation of the delivery system, repositioned the posterior clasp centrally on the P2 scallop in the flail segment, aligning the clasps in a 12 to 6 orientation. All maneuvers were performed under transesophageal echocardiography guidance to ensure no leaflet tension, adequate leaflet release, and no additional MR jets suggestive of valve distortion.

Leaflet optimization was finally performed by individually raising and lowering the clasps to create leaflet slack, ensuring optimal insertion.

**Figure 5:**
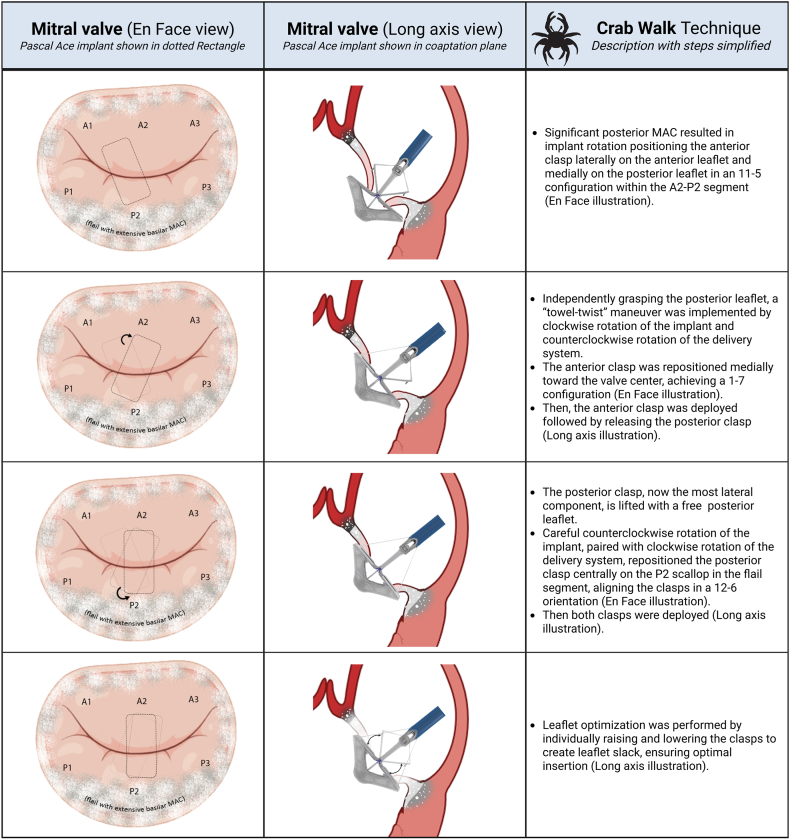
Key figure—Illustration of Crab-Walk technique. Abbreviation: MAC, mitral annular calcification.

Thus, slowly “walking” from nonoptimal leaflets positions, by grasping 1 leaflet and carefully rotating the other clasp after release to grasp the other leaflet in a more optimal position, in small precise steps, could achieve optimal anatomical leaflet grasping. Leaflet optimization step is crucial to ensure adequate leaflet slack and eliminating any tension or distortion of the leaflets (Figure 5; Videos 5-8).

**Figure 6:**
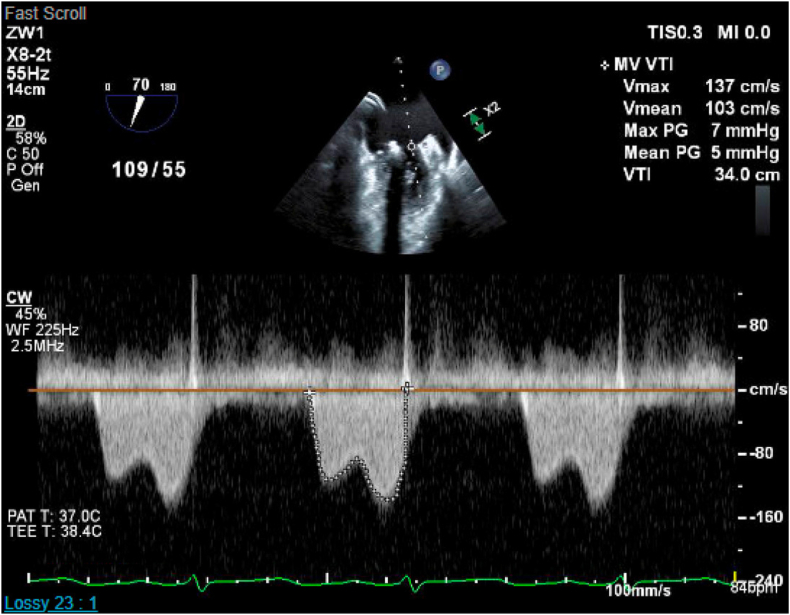
Intraprocedural TEE showing mitral mean gradient of 5 mmHg by CW Doppler before implant release. Abbreviations: CW, continuous wave; MI, magnification; MV, mitral vave; PAT, patient; PG, peak gradient; TEE, transesophageal echocardiography; VTI, velocity time integral.

**Figure 7:**
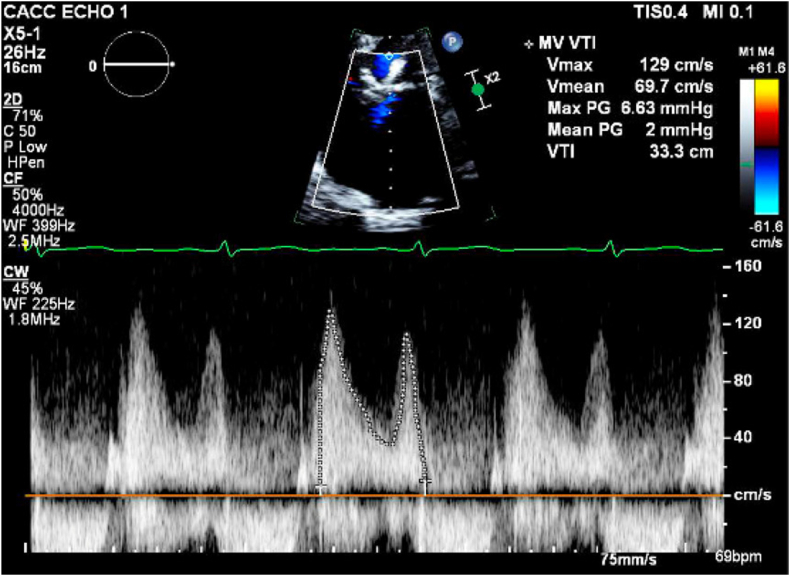
Three-month TTE showing mitral mean gradient of 2 mmHg by CW Doppler. Abbreviations: CF, frequency; CW, continuous wave; MI, magnification; MV, mitral vave; PG, peak gradient; TTE, transesophageal echocardiography; VTI, velocity time integral.

**Figure 8:**
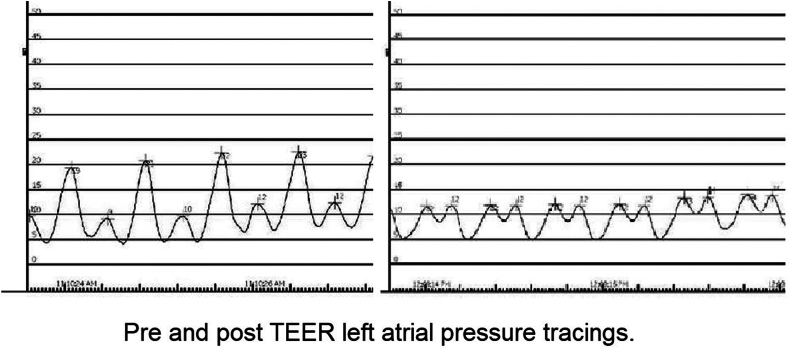
Left atrial pressure tracing pre-TEER and post-TEER. Abbreviation: TEER, transcatheter edge-to-edge repair.

Postprocedure, only mild residual MR (1+) was observed, with a mean gradient of 5 mmHg before implant release and 2 mmHg at 3-month follow-up echocardiography (Figures 6 and 7; Videos 7 and 9). Peak left atrial pressure decreased from 21 to 13 mmHg, with a stable mean pressure of 10 mmHg preprocedure and postprocedure (Figure 8). The patient’s functional status improved significantly; she was weaned off supplemental oxygen and required minimal diuretics.

## Discussion

MAC is a degenerative process associated with cardiovascular risk factors (e.g., hypertension, diabetes, hypercholesterolemia, and chronic kidney disease), female sex, and systemic inflammation.^1^ Its prevalence reaches 33% in patients over 90 years, with an annual incidence of up to 4.7% in those over 75 years.^1^ MAC often leads to mitral valve dysfunction, particularly regurgitation, increasing morbidity and mortality.^1^ Mitral valve surgery in MAC is associated with elevated risks, including atrioventricular groove injury, left circumflex artery injury, conduction disturbances, paravalvular regurgitation, and stroke. Transcatheter interventions also pose challenges, particularly for TMVR, where associated small left ventricular cavities and more acute aortomitral angulation increase the risk of neo-left ventricular outflow tract obstruction, device protrusion, dislodgment, and hemolysis from paravalvular regurgitation is more common in MAC.^1^

Mitral TEER is an established alternative to surgery for high-risk patients with severe MR, but moderate-to-severe MAC patients have been historically excluded from randomized controlled trials.^2^ MAC often extends to the leaflets, complicating device deployment and increasing the risk of high postprocedural gradients and mitral stenosis due to added leaflet tension and smaller valve orifice areas.^3^

The Edwards Lifesciences PASCAL Precision system, a Food and Drug Administration-approved TEER device for degenerative MR, offers advantages in MAC patients. Its horizontal grasping orientation, curved nitinol paddles, and independent leaflet capture minimize traumatic grasping and leaflet tension. The central spacer fills the regurgitant orifice, reducing the leaflet approximation distance.

The Crab Walk technique enhances precise anatomical deployment by allowing independent leaflet grasping, overcoming MAC-related challenges. The technique involves maintaining a capture-ready position with open paddles, staying in attachment to 1 leaflet using the independent clasps, and steering the implant toward the desired leaflet followed by lowering the clasp once leaflet grasp confirmed by 3D imaging. Hence, walking from a more lateral position to a more medial position, in this case, along the coaptation plane of the valve. Optimization is usually performed by withdrawing the implant catheter to create slack, and retracting followed by lowering the clasp slider to achieve optimal grasping.

In this case, the PASCAL system with the Crab Walk technique achieved optimal procedural success despite significant MAC and small mitral valve area (3.8 cm^2^). MR was reduced to mild without significant mitral stenosis, owing to the device’s favorable profile. Although mitral valve TEER is reported to be safe and feasible in patients with more than mild MAC based on several studies,^2^^,^^3^ a mitral valve area <4 cm^2^ was an exclusion criterion in major TEER trials, including the CLASP IID pivotal trial. It is important to mention that this was a last resort technique in a very complex MAC anatomy with small MVOA, and slight technical errors may lead to valve anatomy distortion with incomplete reduction of MR in addition to significant iatrogenic mitral stenosis post-TEER. To our knowledge, this is the first reported case of successful PASCAL TEER using the Crab Walk technique in a patient with a small mitral valve area and significant MAC involving the leaflets.

## Conclusion

Managing severe MR in high surgical risk patients with significant MAC and small mitral valve areas is challenging, particularly when TMVR is not feasible. Tailored TEER with the PASCAL system, leveraging techniques like Crab Walk, offers a viable interventional strategy. Comprehensive device knowledge and procedural expertise at heart valve centers of excellence are critical for success.

## Consent Statement

Consent was obtained.

## Funding

The authors have no funding to report.

## Disclosure Statement

The authors report no conflict of interest.

## Supplementary Material

Video 1 Baseline parasternal TTE showing MAC and severe eccentric MR.

Video 2 Preprocedural mid-esophageal TEE showing MAC, anterior jet MR, and flail P2.

Videos 3 and 4 Preprocedural 3D TEE mitral enface views showing MAC and flail P2.

Video 5 Intraprocedural TEE showing repositioning attempts with difficult leaflet grasp due to MAC.

Video 6 Intraprocedural TEE showing Crab-Walk technique and successful leaflet capture.

Video 7 Pascal device release on fluoroscopy.

Video 8 Intraprocedural TEE showing mild residual MR postimplant.

Video 9 Three-month TTE showing sustained results with mild residual MR.
